# Synergistic potential of teriflunomide with fluconazole against resistant *Candida albicans in vitro* and *in vivo*

**DOI:** 10.3389/fcimb.2023.1282320

**Published:** 2023-12-19

**Authors:** Xiuyun Li, Bing Kong, Yaqiong Sun, Fenghua Sun, Huijun Yang, Shicun Zheng

**Affiliations:** ^1^ Maternal and Child Health Development Research Center, Shandong Provincial Maternal and Child Health Care Hospital Affiliated to Qingdao University, Jinan, Shandong, China; ^2^ Department of Natural Product Chemistry, Key Laboratory of Chemical Biology (Ministry of Education), School of Pharmaceutical Sciences, Cheeloo College of Medicine, Shandong University, Jinan, Shandong, China; ^3^ Department of Critical Care Medicine, Shandong Provincial Maternal and Child Health Care Hospital Affiliated to Qingdao University, Jinan, Shandong, China; ^4^ Obstetrics Department, Shandong Provincial Maternal and Child Health Care Hospital Affiliated to Qingdao University, Jinan, Shandong, China; ^5^ Radiology Department, Shandong Provincial Maternal and Child Health Care Hospital Affiliated to Qingdao University, Jinan, Shandong, China; ^6^ Reproductive Medicine Center, Shandong Provincial Maternal and Child Health Care Hospital Affiliated to Qingdao University, Jinan, Shandong, China

**Keywords:** teriflunomide, *Candida albicans*, fluconazole, drug combination, *Galleria mellonella*

## Abstract

**Introduction:**

*Candida albicans* is the primary cause of systemic candidiasis, which is involved in high morbidity and mortality. Drug resistance exacerbates these problems. In addition, there are limited antifungal drugs available. In order to solve these problems, combination therapy has aroused great interest. Teriflunomide is an immunosuppressant. In the present work, we aimed to identify whether teriflunomide can reverse the resistance of *Candida albicans* in the presence of sub-inhibitory concentrations of fluconazole *in vitro* and *in vivo*.

**Methods:**

Seven *Candida albicans* isolates were used in this study. Susceptibility of *Candida albicans in vitro* to the drugs was determined using a checkerboard microdilution assay in accordance with the recommendations of the Clinical and Laboratory Standards Institute. The effects of drugs on biofilm biomass of *Candida albicans* were determined by crystal violet staining. The development ability of *Candida albicans* hyphae was performed using a modified broth microdilution method. *Galleria mellonella* was used for testing the *in vivo* efficacy of the combination therapies.

**Results:**

We found that the combination of teriflunomide (64 µg/mL) and fluconazole (0.5-1 µg/mL) has a significant synergistic effect in all resistant *Candida albicans* isolates (n=4). Also, this drug combination could inhibit the immature biofilm biomass and hyphae formation of resistant *Candida albicans*. *Galleria mellonella* was used for testing the in vivo efficacy of this combination therapies. As for the *Galleria mellonella* larvae infected by resistant *Candida albicans*, teriflunomide (1.6 µg/larvae) combined with fluconazole (1.6 µg/larvae) significantly increased their survival rates, and reduced the fungal burden, as well as damage of tissue in comparison to that in the control group or drug monotherapy group.

**Conclusion:**

These results expand our knowledge about the antifungal potential of teriflunomide as an adjuvant of existing antifungal drugs, and also open new perspectives in the treatment of resistant *Candida albicans* based on repurposing clinically available nonantifungal drugs.

## Background

Invasive *Candida* bloodstream infections remain the most frequent life-threatening fungal disease, with *Candida albicans* (*C. albicans*) isolates accounting for 70% to 80% of the *Candida* isolates isolated from infected patients (Chin et al., 2016). Indeed, *C. albicans* bloodstream infections cause a mortality rate more than 40% (Pfaller and Diekema, 2007; Gow and Yadav, 2017; Robbins et al., 2017). Moreover, the broad and irrational utilization of azole antifungals, especially fluconazole, has led to the emergence azole-resistant clinical isolates of *C. albicans* (Kohli et al., 2002). Owing to the limited number of available antifungal drugs in clinic, there is a need for alternative approaches or developing new antifungal drugs against resistant *C. albicans* infections.

The development of new drugs is an expensive and time-consuming project (Khanna, 2012; Wang et al., 2016). Only one antifungal drug (isavuconazole) was newly licensed in the last ten years (Van Daele et al., 2019). The lack of new antifungal drugs and the continual buildup of resistance mechanisms by *C. albicans* require new strategies with satisfying antifungal efficacy (Fisher et al., 2022). Combination drug therapy may be an effective way to overcome resistant fungi. In fact, drug combinations have already been used to treat many diseases, such as cancer, HIV or cardiovascular disease (Lehar et al., 2009; Ahmad et al., 2015; Shrestha et al., 2015). Combining a new antifungal agent with a known antifungal drug, with a different or similar mechanism of action, would represent a novel therapeutic approach, which may circumvent the drug resistance problem. To date, there have been many studies of FDA-approved drugs used in combination with existing antifungal drugs to overcome resistant *C. albicans*, the rationale being that the safety of FDA-approved drugs has been studied and their properties are well known (Li et al., 2019b; Rajasekharan et al., 2019; Zhang et al., 2020).

Teriflunomide, a dihydrowhey acid dehydrogenase inhibitor, is an FDA-approved immunomodulator with low cytotoxicity, and used to treat relapse-remission multiple sclerosis in the clinic (Wiese et al., 2013; Scott, 2019; Barua et al., 2023; Lang et al., 2023). Up to now, no study on the antifungal activity of teriflunomide has so far been reported. Inspired by the antifungal effects of other immunomodulators, such as tacrolimus and ciclosporin A, we attempt to explore whether teriflunomide can be used in combination with azole antifungal drugs to combat resistant *C. albicans*. Fluconazole is an azole antifungal drug with low toxicity and high bioavailability. We aimed to identify whether teriflunomide could potentially enhance its antifungal activity. For this purpose, the synergistic antifungal activity of fluconazole combined with teriflunomide on *C. albicans* were investigated *in vitro* and *in vivo*. Furthermore, we also attempted to explore the potential antifungal mechanism for this drug combination.

## Materials and methods

### Microorganisms and drug preparation

Seven isolates of *C. albicans* (SC5314, CA4, CA8, CA10, CA16, CA103 and CA137) were used in this study, which were isolated from the blood of patients and kindly provided by Professor Shujuan Sun (Shandong Provincial Qianfoshan Hospital, Jinan, China). *C. albicans* ATCC 10231 was used as a reference strain of the study. All isolates were taken from previously established stocks. Before all experiments, isolates were routinely inoculated twice in yeast extract-peptone-dextrose agar medium overnight at 35°C. Teriflunomide (Dalian Meilun Biotech Co., Ltd, Dalian, China) was dissolved in ethyl alcohol at a concentration of 54, 000 μg/mL, whereas fluconazole (Dalian Meilun Biotech Co., Ltd, Dalian, China) was dissolved in sterilized water at a concentration of 5, 120 μg/mL. To avoid affecting the outcomes of experiments, the proportion of ethyl alcohol was less than 1% of the whole test volume, according to the Clinical and Laboratory Standards Institute M27-A3 (CLSI M27-A3) (Rex et al., 2008). This study was approved by the Scientific Research Ethics Committee of Shandong Provincial Maternal and Child Health Hospital (No. 2023-083).

### Determination of antifungal activity on planktonic cells

Susceptibility of planktonic cells of *C. albicans* to the drugs was determined using a checkerboard microdilution assay in accordance with the recommendations of CLSI M27-A3 (Rex et al., 2008). The minimal inhibitory concentrations (MIC) of teriflunomide and fluconazole against *C. albicans* strains were determined by broth microdilution method as described previously (Mo et al., 2020). Yeast with a final concentration of 1 ×10^3^ cells/mL was inoculated in the RPMI 1640 liquid medium with serial (2 ×) dilutions of each drug on 96-well flat bottom plates. After incubation at 35 °C for 24 h, MIC was visually determined as the lowest concentration of drug that reduced ≥50% growth in comparison to growth control without drug treatment (Ernst et al., 2002; Rex et al., 2008). Fractional inhibitory concentration (FIC) indexes (FICI) model was applied for the calculation of each combination using an equation, FIC_A_ + FIC_B_ = FICI, where FIC_A_ is the MIC of fluconazole in combination divided by the MIC of fluconazole alone, and FIC_B_ is the MIC of teriflunomide in combination divided by the MIC of teriflunomide alone. The results were defined as FICI ≤ 0.5 for synergism, FICI > 4.0 for antagonism, and 0.5 < FICI ≤ 4.0 for no interaction (Odds, 2003).

### Biofilm biomass production

To further assess the synergistic antifungal mechanisms, we tested the effect of teriflunomide in combination with fluconazole against the biofilm biomass of resistant *C. albicans* (CA10). Living and dead fungal cells were stained with crystal violet (Petrachi et al., 2017). The total biomass of biofilms was assessed using crystal violet staining, with slight modifications (Manoharan et al., 2017). Cell suspensions were adjusted to reach a concentration of 1 × 10^6^ CFU per mL in RPMI 1640 medium. Then, 200 μL of cell suspensions was transferred into 96-well flat bottom plates and incubated for different times (4 h, 24 h) at 37°C. After incubation, non-adhered cells were removed by washing thrice with sterile PBS, and then 200 μL of desired concentration of drugs in RPMI 1640 medium was added to each well of 96-well flat bottom plates. The plates were incubated at 37°C for 24 h to evaluate the effect of drugs on biofilm biomass by crystal violet (0.1%) staining. Briefly, wells were washed twice with sterile PBS to remove all non-adherent cells and then stained with crystal violet for 5 min. The wells were washed three times with sterile PBS. After drying, 120 μL of 95% ethanol was added into each well. After 10 min of 95% ethanol treatment, 100 μL of suspension from each well was transferred to a new 96-well flat bottom plate and the optical density (OD) values at 570 nm was measured using a microplate reader (Epoch2, Agilen BioTek Co., Ltd., USA). Each experiment was performed at least three times.

### Hyphal morphology

Yeast-to-hyphae phenotype switching is a characteristic pathogenic trait of *C. albicans* (Boyce and Andrianopoulos, 2015). The hyphal development ability was performed using a modified broth microdilution method (Chang et al., 2012). Both 100 μL of *C. albicans* (CA10) cell suspensions (2 × 10 ^5^ CFU per mL) and 100 μL of desired concentration of drugs in RPMI 1640 medium was simultaneously added to each well of 96-well flat bottom plates. Wells containing *C. albicans* and RPMI 1640 alone served as controls. The plates were incubated at 35°C for 4 h. After incubation, cells were observed for morphological transition under a fluorescent microscope (ECLIPSE Ts2, Nikon Instruments, Japan). Each experiment was performed at least three times.

### Survival assay of *Galleria mellonella*

*Galleria mellonella* (*G. mellonella*) larvae were used as an invertebrate infection model to evaluate the *in vivo* interactions between fluconazole and teriflunomide, as described previously with some modifications (Li et al., 2019a). Four groups of eighteen randomly chosen larvae with a similar weight (ca. 0.20 g) and injected with 10 μL of a yeast (CA10) suspension (1 × 10^8^ CFU/mL) via the last left proleg. After incubation at 35 °C for 2 h, each larva was injected with 10 μL of sterile PBS, fluconazole (160 μg/mL), teriflunomide (160 μg/mL), or fluconazole (160 μg/mL) plus teriflunomide (160 μg/mL) via the last right proleg. All larvae were incubated at 35 °C in the dark, and the numbers of *G. mellonella* that survived were recorded daily for a period of 4 days. The larvae not responding to touch were considered dead. The survival curves were plotted by the Kaplan-Meier method using SPSS 22 software. Each experiment was performed at least three times.

### Histological study of *G. mellonella*

For histological study, four groups of eighteen randomly chosen larvae with a similar weight (ca. 0.20 g) and the experimental procedures were the same as described above. Three larvae incubated for two days were randomly selected from each group and cut into sections (7 μm). Sections stained with periodic acid Schiff (PAS) stain were observed using the digital slice scanning system (Precice 510, Unicmedical equipment Co., Ltd., China). Each experiment was performed at least three times.

## Results

### Teriflunomide in combination with fluconazole synergistically inhibited the cell growth of resistant *C. albicans in vitro*

The combination of teriflunomide and fluconazole was found to have a significant synergistic effect against all resistant *C. albicans* isolates (100%) in this study (**Table 1**). When fluconazole was combined with teriflunomide (64 µg/mL), the MIC ranges of fluconazole decreased from >512 µg/mL to 0.5-1 µg/mL, suggesting a strong synergy for the combination of teriflunomide and fluconazole against resistant *C. albicans*. No synergistic effect was found for this drug combination against susceptible *C. albicans* isolates (**Table 1**).

**Table 1:** Drug interactions of fluconazole and teriflunomide against *C. albicans in vitro*.

Drugs	Isolates ^c^	MIC (μg/mL)^d^				FICI model	
		FLC	FLC_comb_	TER	TER_comb_	FICI^e^	Interpretation
FLC^a^ + TER^b^	SC5314 (S)	2	2	>512	>512	2.0000	no interaction
	CA4 (S)	1	1	>512	>512	2.0000	no interaction
	CA8 (S)	1	1	>512	>512	2.0000	no interaction
	CA10 (R)	>512	0.5	>512	64	0.1260	synergism
	CA16 (R)	>512	0.5	>512	64	0.1260	synergism
	CA103 (R)	>512	1	>512	64	0.1270	synergism
	CA137 (R)	>512	1	>512	64	0.1270	synergism

a FLC: fluconazole.

b TER: teriflunomide.

c S: susceptible; R: resistant.

d MIC was considered as the lowest concentration of drug that reduced ≥50% cell growth in comparison to cell growth control without drug treatment.

e FICI ≤ 0.5 for synergism, 0.5 < FICI ≤ 4.0 for no interaction.

### Teriflunomide in combination with fluconazole inhibited biomass of immature biofilm

**Figure 1:**
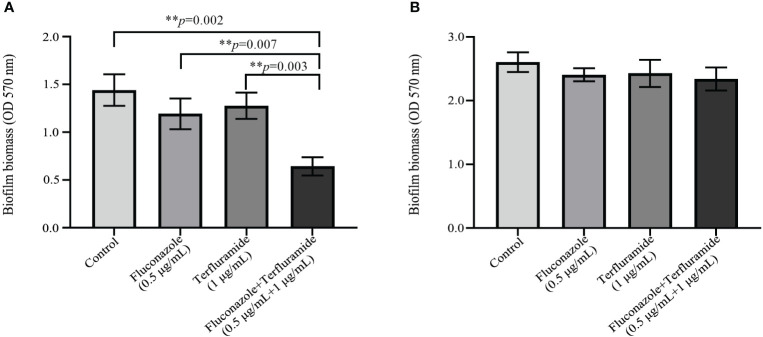
Teriflunomide in combination with fluconazole inhibited the biofilm biomass. **(A)** immature biofilm (4 h); **(B)** mature biofilm (24 h).

Crystal violet staining is often used for the determination of yeast biofilm biomass, mainly because of its high detection accuracy for a large number of biofilms (Kulisova et al., 2023). **Figure 1** showed the effect of teriflunomide in combination with fluconazole on the immature or mature biofilm biomass of resistant *C. albicans*. Compared with the control group, fluconazole group alone, or teriflunomide group alone respectively, the biomass of immature biofilm (4 h) was significantly inhibited by this drug combination (**Figure 1A**). However, compared with the other three groups, the biomass of mature biofilm (24 h) was not significantly inhibited by this drug combination (**Figure 1B**).

### Teriflunomide in combination with fluconazole inhibited yeast-to-hypha morphological transition

**Figure 2:**
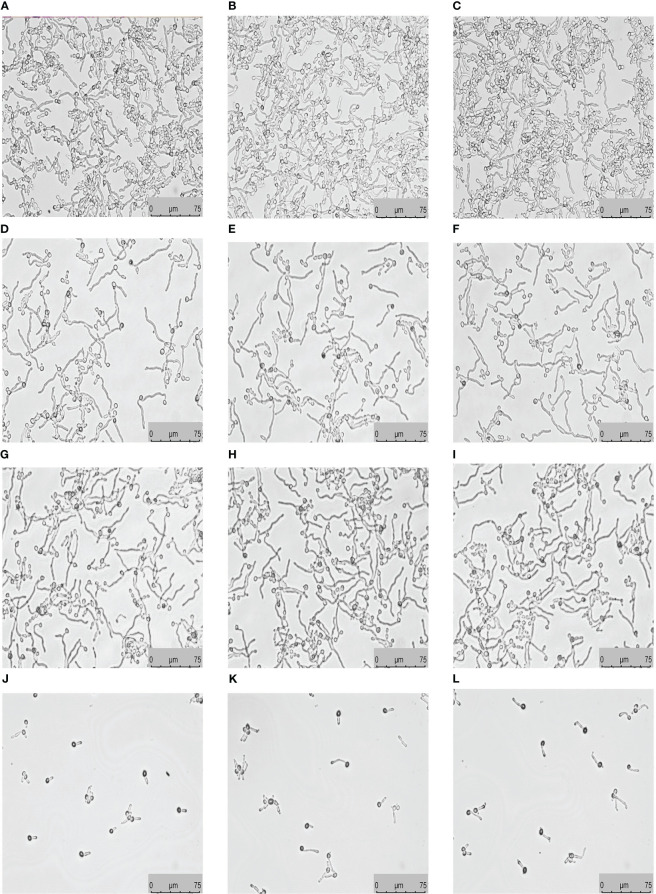
Teriflunomide in combination with fluconazole inhibited yeast-to-hypha morphological transition. **(A–C)**: control; **(D–F)**: fluconazole (1 µg/mL); **(G–I)**: teriflunomide (64 µg/mL); **(J–L)**: teriflunomide (64 µg/mL) in combination with fluconazole (1 µg/mL).

Liquid RPMI 1640 medium is known to induce morphological transition of *C. albicans*. To determine the effect of teriflunomide alone or in combination with fluconazole on the yeast-to-hypha morphological transition of resistant *C. albicans*, CA10 cells and drugs were placed in liquid RPMI 1640 medium. *C. albicans* in the control group without drug treatment revealed the formation of long true hyphae (**Figures 2A–C**). The teriflunomide and fluconazole alone group showed the similar amounts of hyphae as the control group (**Figures 2D–I**). Interestingly, hyphae were scarcely observed in the group of teriflunomide (64 µg/mL) in combination with fluconazole (1 µg/mL), indicating this drug combination could inhibit the yeast-to-hypha morphological transition of resistant *C. albicans* (**Figures 2J–L**).

### Teriflunomide in combination with fluconazole showed good efficacy on resistant *C. albicans in vivo*

**Figure 3:**
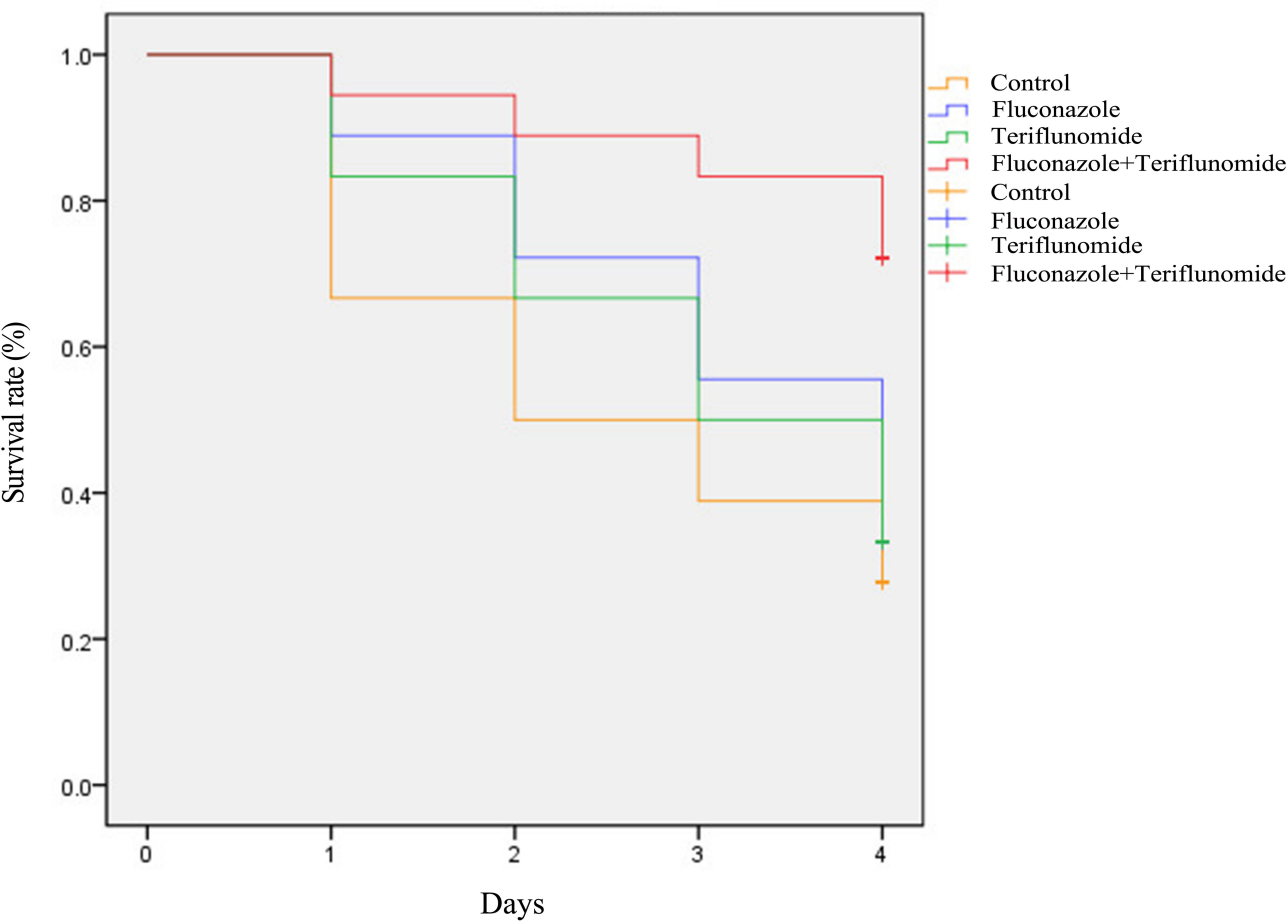
Teriflunomide in combination with fluconazole improved the survival rates of *G. mellonella* after infection. A log-rank test for these curves was conducted and the p value was 0.024.

In the *in vivo* experiment, eighteen randomly chosen *G. mellonella* larvae in each group were injected with the CA10 suspension, and after 2 h of infection, the larvae were treated with drugs. As can been seen from **Figure 3**, teriflunomide combined with fluconazole kept the larvae free from CA10 infections and resulted in significantly higher survival of the larvae over a 4-day infection. In brief, the mean survival rate of larvae on the 2nd-4th day in the control group, fluconazole group and teriflunomide group was 31-50%, 35-70% and 33-69% respectively (**Supplementary Table 1**). Notably, the survival rate of larvae on 2nd-4th in the drug combination group was 69-87%, which was significantly higher than that of the control group or drug monotherapy group (*p* < 0.05) (**Supplementary Table 1**). Data from any one experiment were shown in **Figure 3**, which indicated that the combination of teriflunomide and fluconazole significantly increased the survival rates of infected larvae.

**Figure 4:**
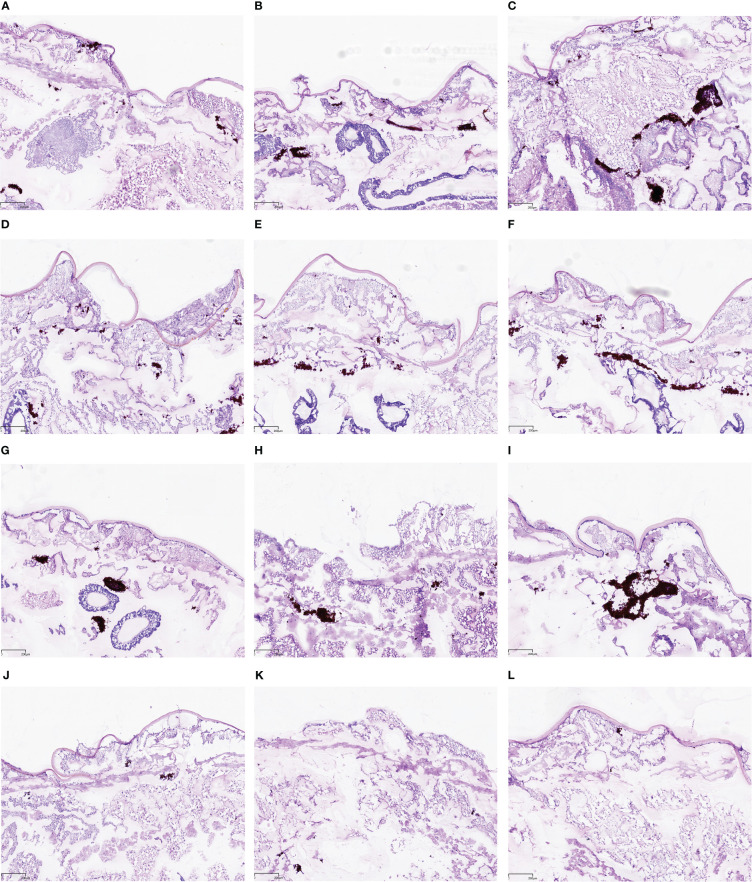
Teriflunomide in combination with fluconazole reduced the fungal burden and damage in tissues of *G. mellonella* after infection. **(A–C)**: control; **(D–F)**: fluconazole; **(G–I)**: teriflunomide; **(J–L)**: teriflunomide in combination with fluconazole.

Regarding observation of histological sections (**Figure 4**), the infected tissues showed black areas after PAS staining, and the black areas contained clustered yeast cells and hyphae. As can been seen from **Figure 4**, black areas in the fluconazole-monotherapy group and teriflunomide-monotherapy group as well as the control group were numerous and large, while those in drug combination group were obviously much fewer and smaller. These observations suggested that teriflunomide combined with fluconazole significantly reduced the fungal burden and tissue damage of the resistant C. albicans to the larvae in comparison with that in the control group or drug monotherapy group.

## Discussion

In the last decades, *C. albicans* has served as the leading causal agent of life-threatening invasive infections with mortality rates approaching 40% despite treatment (Chen et al., 2020). Besides the high mortality, resistance of *C. albicans* to conventional antifungal drugs is also the paramount concern in the field of medical mycology. The emergence of drug-resistant isolates of *C. albicans* has created a higher risk for clinical infections and is a growing concern. Because none of the current antifungals have all the characteristics of the ideal antifungals, and the discovery rate of new antifungals is declining, the study of drug combination against drug-resistant fungal infections is receiving increasing attention (Kaneko et al., 2013; Wong et al., 2014). In recent years, there has been increasing interest in repurposing FDA-approved drugs because their clinical and toxicological properties are already known, thus reducing the cost and time of drug development. Many FDA-approved drugs from pharmacologically distinct families have been proved to have antifungal potentials against resistant *C. albicans*, such as aripiprazole, ambroxol hydrochloride, and ribavirin (Li et al., 2019b; Rajasekharan et al., 2019; Zhang et al., 2020). Inspired by these findings, we evaluated the interaction of teriflunomide, an FDA-approved immunomodulator with low cytotoxicity, and fluconazole against resistant *C. albicans*. In this study, no synergistic effect was found for this drug combination against the susceptible *C. albicans* isolates (**Table 1**). Nevertheless, we first found that teriflunomide can reverse the resistance of *C. albicans* to fluconazole *in vitro* (**Table 1**). The combination of teriflunomide (64 µg/mL) and fluconazole (0.5-1 µg/mL) was found to have strong synergistic effects against planktonic cells of four resistant *C. albicans* isolates, demonstrating the antifungal potentials of this drug combination.

To clarify the synergistic effects of this drug combination against resistant *C. albicans*, further exploration of the underlying mechanisms is quite needed. Many mechanisms seem to play important roles in the development of *C. albicans* resistance. Among them, over-expression of efflux pumps, biofilm, and hypha are the most important research hotspots. So far, several drug combinations have been proved to exert their synergistic antifungal effects against resistant *C. albicans* by inhibiting the over-expression of efflux pumps (Usai et al., 2019; Lu et al., 2020). What’s more, biofilm formation of *C. albicans* is among the culprits of its resistance, with multiple studies reporting up to a 1000-fold greater drug resistance in biofilm-forming cells compared with non-biofilm cells *in vitro* (Tobudic et al., 2012). Many available antifungal drugs against *C. albicans* are capable of controlling the growth of planktonic cells alone, resulting in their invalidation in controlling biofilms *in vivo*, which eventually may lead to the therapeutic failure in a clinical scenario (Taff et al., 2013). Different *C. albicans* morphotypes differentially elicit host immune responses and the production of cytokines. Hyphae, which represent an important phase of *C. albicans* in the disease process, can cause tissue damage by invading mucosal epithelial cells (Chen et al., 2020). The formation of hyphae ultimately forms biofilms, which protect sessile yeast cells from antifungal drugs and may induce new infections. While drug resistance mechanisms in the commensal human pathogen *C. albicans* are continually evolving, hyphae are still one of the most important factors associated with drug resistance. Therefore, exploring better therapeutic therapies to combat the hyphae of *C. albicans* appears to be particularly vital. Therefore, we further studied the efflux pump activity, biofilm biomass, and hyphae formation of resistant *C. albicans* isolates with the treatment of this drug combination. The present study found that this drug combination has no impact on the activity of efflux pump activity (**Supplementary Figure 1**). However, teriflunomide in combination with fluconazole can synergistically inhibit the biomass of resistant *C. albicans* immature biofilm (4 h) (**Figure 1A**). Our results in this study also indicated that teriflunomide in combined with fluconazole exerts the synergistic effects against resistant *C. albicans* by inhibiting its hyphae formation (**Figure 2**). Furthermore, this study not only provides a theoretical basis for the identification of targets in candidiasis treatment, but also gives some reference to the study of novel antifungal drugs.

*G. mellonella* is a convenient *in vivo* model for assessing the activity and toxicity of antimicrobial agents and for studying the immune response to pathogens and provide results similar to those from mammals (Brennan et al., 2002; Kavanagh and Sheehan, 2018; Jemel et al., 2020). *G. mellonella* larvae are now widely used in academia and their use can assist in the identification and evaluation of novel antimicrobial agents. *G. mellonella* larvae are inexpensive to purchase and house, easy to inoculate, generate results within 24–48 h and their use is not restricted by legal or ethical considerations. In this study, the *in vivo* antifungal effects of teriflunomide combined with fluconazole was evaluated by using the model of *G. mellonella*. The determination of survival curve showed that teriflunomide combined with fluconazole was very effective in protecting larvae from fatal infection by resistant *C. albicans* (**Figure 3**). In addition, histological examination plays an important role in studying the virulence of infection. In this study, it showed that the virulence was related to the degree of tissue damage. Resistant *C. albicans* produced hyphae and induced serious tissue damage in larvae. Many infected black areas and clusters of yeast cells were also observed. After the treatment of drug combination, fewer clustered yeast cells and hyphae were observed (**Figure 4**). The good efficacy of the combination therapies of teriflunomide combined with fluconazole on *G. mellonella* infected by resistant *C. albicans* was confirmed.

## Conclusion

In conclusion, our findings demonstrated a potential use of this drug combination in prevention or early treatment of resistant *C. albicans* infections. Inhibition the biomass of immature biofilm and hyphae formation provided an explanation of the synergistic mechanisms for this drug combination. Although teriflunomide has a certain degree of side effects in clinical use, with the modification of the structure of teriflunomide, this combination or their analogues may become a new alternative way to treat resistant C. albicans infections. Besides, this study on the repurposing of teriflunomide could also serve as an example to inspire the reapplication of other FDA-approved drugs in the antifungal field.

## Data availability statement

The original contributions presented in the study are included in the article/**Supplementary Material**. Further inquiries can be directed to the corresponding authors.

## Ethics statement

This study was approved by the Scientific Research Ethics Committee of Shandong Provincial Maternal and Child Health Hospital (No.2023-083).

## Author contributions

XL: Conceptualization, Data curation, Methodology, Writing – original draft. BK: Conceptualization, Data curation, Writing – original draft. YS: Conceptualization, Data curation, Writing – original draft. FS: Conceptualization, Writing – original draft. HY: Conceptualization, Writing – review & editing. SZ: Conceptualization, Writing – review & editing.
