# Supplementary material for: Synergistic potential of teriflunomide with fluconazole against resistant Candida albicans in vitro and in vivo
Source: Front Cell Infect Microbiol. 2023 Dec 19;13:1282320. doi: 10.3389/fcimb.2023.1282320 (PMC10758495; doi:10.3389/fcimb.2023.1282320)
Supplement: Supplementary file 1 [file Image_1.pdf]

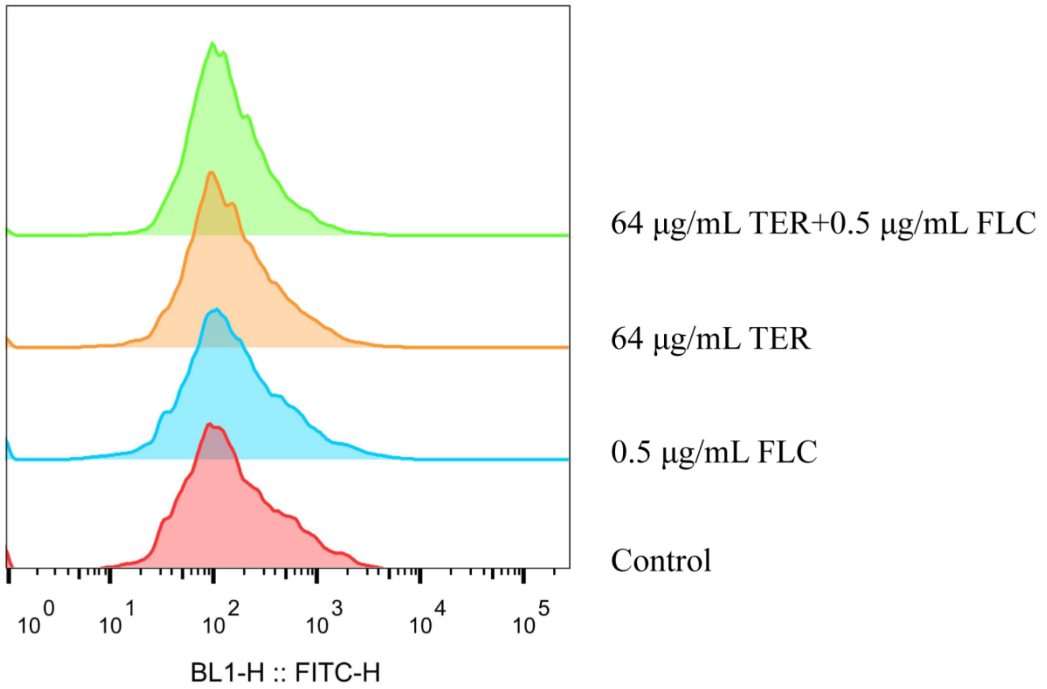

Supplementary Figure 1 Effects of TER in combined with FLC on the efflux of rhodamine 123 of CA10.

The effect of drugs on efflux pump in CA10 was determined by rhodamine 123 (Rh123). Specifically, after adjusting the log-growing CA10 cells to obtain a fungal suspension (1×10^6 CFU/mL) with glucose-free PBS, the cells were incubated at 35°C for 4 h to deplete their energy. Rh123 fluorescent probe at a final concentration of 5 μM was added to the energy depleted CA10 cells for 30 min staining, and then cells were washed with PBS three times to remove the unbound Rh123. 200 μL cell suspension was taken from each group, and the mean fluorescence intensity of each group before dosing was measured by flow cytometry (excitation wavelength 507 nm, emission wavelength 530 nm). The remaining cells were then suspended into a glucose-containing PBS to initiate efflux pump activity and simultaneously exposed to different drugs. After incubation for 30 min, the cells of each group were centrifuged and cleaned, and then the mean fluorescence intensity of Rh123 was determined by flow cytometry (excitation wavelength 507 nm, emission wavelength 530 nm). The difference of mean fluorescence intensity before and after drug treatment is considered to be the strength of drugs' influence on efflux pump. FlowJo 10.0 software was used to analyze the data.
