# Supplementary material for: Synergistic potential of teriflunomide with fluconazole against resistant Candida albicans in vitro and in vivo
Source: Front Cell Infect Microbiol. 2023 Dec 19;13:1282320. doi: 10.3389/fcimb.2023.1282320 (PMC10758495; doi:10.3389/fcimb.2023.1282320)
Supplement: Supplementary file 2 [file Table_1.doc]

**Supplementary Table 1 Survival situation of infected *Galleria mellonella* from three separate experiments**

| **Days** | **Uninfected Larvae** | **Control** | **Fluconazole** | **Teriflunomide** | **Fluconazole**  **+**  **Teriflunomide** | ***p* values** |
| --- | --- | --- | --- | --- | --- | --- |
| 1 | **18**, 18, 18 | **12**, 11, 13 | **16**, 17, 14 | **15**, 16, 13 | **17**, 17, 16 | *p_a_*=0.010;  *p*_b_=1.000;  *p_c_*=0.483 |
| 2 | **18**, 18, 18 | **9**, 8, 10  (mean survival rate, 50%) | **13**, 14, 11  (mean survival rate, 70%) | **12**, 13, 12  (mean survival rate, 69%) | **16**, 16, 15  (mean survival rate, 87%) | ***p_a_*＜0.001;**  ***p*_b_=0.038;**  ***p_c_*=0.021** |
| 3 | **18**, 18, 18 | **7**, 6, 8  (mean survival rate, 39%) | **10**, 11, 10 (mean survival rate, 57%) | **9**, 10, 8  (mean survival rate, 50%) | **15**, 14, 14  (mean survival rate, 80%) | ***p_a_*＜0.001;**  ***p*_b_=0.002;**  ***p_c_*＜0.001** |
| 4 | **18**, 18, 18 | **5**, 5, 7  (mean survival rate, 31%) | **6**, 7, 6  (mean survival rate, 35%) | **6**, 6, 6  (mean survival rate, 33%) | **13**, 12, 12  (mean survival rate, 69%) | ***p_a_*＜0.001;**  ***p*_b_＜0.001;**  ***p_c_*＜0.001** |

*p_a_*: *p* value of drug combination group compared with control group；

*p*_b_: *p* value of drug combination group compared with fluconazole group；

*p*_c_: *p* value of drug combination group compared with teriflunomide group;

The first experimental data (bolded data) was randomly selected for drawing the survival curve of Figure 3 in the text.
